# Gelatin‐Based Head Phantoms: A Practical Guide for Artificial Brain Signal Research

**DOI:** 10.1155/ijbm/2255123

**Published:** 2026-04-29

**Authors:** Elif Nur Selçuk, Gökçenur Çakmak, Mustafa Reşit Usal

**Affiliations:** ^1^ Department of Mechanical Engineering, Graduate School of Natural and Applied Sciences, Suleyman Demirel University, Isparta, Turkey, sdu.edu.tr; ^2^ Department of Mechanical Engineering, Faculty of Engineering and Natural Sciences, Suleyman Demirel University, Isparta, Turkey, sdu.edu.tr

**Keywords:** artificial brain wave, electroencephalography, tissue mimicking phantoms

## Abstract

**Background:**

The involvement of human subjects in the development of biomedical devices presents both ethical and practical challenges. Electroencephalography (EEG) signals exhibit interindividual variability and are subject to fluctuations induced by movement and emotional states. Hence, the fabrication of artificial tissues (phantoms) capable of accurately replicating human organs and tissues is of critical importance. The primary objective of this study was to create phantoms that accurately mimic the electrical conductivity of human head tissues.

**Methods:**

Phantom compositions were optimized to accomplish these objectives. This article details the fabrication and characterization of 116 tissue‐mimicking rat head size phantoms (RHSPs) with diverse concentrations, mixing durations, volumes, and combinations of gelatin, salt, reduced graphene oxide (rGO) solution, silver nanopowder (Ag), graphite powder (Gr), polyvinyl alcohol (PVA) solution, sodium alginate (SA) solution, PVA/SA solutions, and potassium sorbate (KS), evaluated for their electrical conductivity properties using an LCR meter. Using the electrical conductivity values derived from the RHSP data, a regression equation was developed in Python, which was then employed to fabricate a human head phantom (HHP).

**Results:**

Conductive polymer–based phantoms with electrical conductivity and biological properties comparable to real cranial tissues were successfully developed, making them suitable for EEG electrode and cap applications. The developed HHP was powered by a signal generator, and artificial EEG brain waves were generated using the OpenBCI platform. Based on the acquired data, brain simulations were conducted using the low‐resolution electromagnetic tomography (LORETA) program. The trials produced phantoms with electrical conductivity consistent with that of most tissues within the layers of the human skull. The study provides a framework for the economical and efficient fabrication of both single‐ and multilayer head phantoms.

## 1. Introduction

Electroencephalography (EEG) monitors cerebral activity noninvasively. Researchers rely on head phantoms that accurately replicate both electrical and anatomical properties to evaluate EEG system performance and optimize electrode configurations [1]. These phantoms create controlled, reproducible experimental environments, avoid ethical constraints, and enable standardized research [2–4]. Scientists have successfully developed specialized phantoms for various tissues, including the skull [5], brain [6, 7], breast [8–10], clavicle [11], and liver [12]. Recent studies have focused on gelatin‐based brain models to investigate tissue‐equivalent electrical behavior [13] and have performed detailed electrical comparisons between these synthetic models and ex vivo porcine skin [14]. Furthermore, 3D‐printed head phantoms have been developed for electrical impedance tomography (EIT), featuring anatomically realistic geometry and continuously varying skull resistivity distributions to better mimic human head impedance [15]. Advanced multilayered electromagnetic phantoms now allow for the investigation of signal propagation through various cranial tissues [16], while 3D printing technology enables the fabrication of functional structures with precise dielectric properties for medical applications [17, 18].

Researchers fabricate these phantoms in solid or liquid forms using methods such as three‐dimensional (3D) printing and casting. Scientists commonly employ liquid phantoms in antenna design due to their saline content, which provides tissue‐like conductivity; however, these phantoms often lack anatomical realism and suffer from high noise and low signal‐to‐noise ratios (SNRs) [19]. Traditionally, researchers developed skull phantoms to study hard tissue electrical properties, while soft tissue simulation remained rare. Early electrophysiological phantoms included melons, which served as primitive solid models due to their size and electrical similarity to the human head [20]. Modern skull phantom technology now spans from simple spherical shapes to anatomically accurate human skull models [21–23]. Researchers increasingly prefer these phantoms over animal cadavers, as laboratories can produce them on‐demand and control their electrical properties more precisely [24, 25].

Layered and customizable phantoms, such as the five‐layer reconfigurable head phantom, improve electrical realism by assigning layer‐specific conductivity values and adapting to diverse experimental conditions [26]. Print‐based phantoms with anisotropic conductivity simulate directional variations in brain tissue, demonstrating the impact of propagation direction on SNR [27]. Although anatomically accurate 3D‐printed human head phantoms (HHPs) and positron emission tomography‐magnetic resonance imaging (PET‐MRI)–compatible models replicate complex head geometry, they may not fully capture frequency‐dependent electrical properties [28–30].

The structure of the skin, brain, and cardiac activity varies among individuals, and signals from the same individual may fluctuate over time [31]. EEG is used to assess brain activity, with typical signal amplitudes ranging from 10 to 100 μV and frequencies between 1 and 100 Hz [32–34]. To capture these physiological signals, researchers place electrodes directly on the skin. Because brain waves are highly sensitive to rapid fluctuations and noise, developing mobile EEG systems, wearable technologies, brain–computer interfaces (BCIs), and low‐cost EEG devices depends closely on advances in electrode design, electrode placement systems (e.g., headcaps), and noise reduction strategies.

Physiological signals vary inherently, requiring large sample sizes when testing these devices on humans [35–37]. To achieve accurate and reliable results in phantom‐based studies, models must closely replicate the geometry and resistivity distribution of a real human cranium. However, developing electrodes for EEG, electrocardiography (ECG), and electromyography (EMG) does not always require full‐scale skull geometry [38]. Testing electrodes on phantoms that mimic the dimensions of small animals—such as a hemispherical rat head with a diameter of 16.8 mm—offers considerable time and cost advantages [39, 40]. The main differences between rat head‐sized phantoms (RHSPs) and HHPs involve shape, size, and the feasibility of invasive electrode placement [41]. Since RHSPs and HHPs share comparable electrical properties, variations in size and shape do not significantly affect experimental outcomes [42]. Skin, as one of the most resistive tissues in the human body, provides a critical reference for EEG measurements and electrode testing [43].

Researchers widely use polyvinyl alcohol (PVA) solutions and PVA–gelatin composites to replicate the frequency‐dependent dynamic behavior of analog signals generated by the human brain [44, 45]. By adjusting mixing ratios, they tailor these materials to achieve bioequivalent properties [46–48]. Brain phantoms based on PVA and barium sulfate exhibit mechanical characteristics comparable to human and porcine skin [15]. Furthermore, gelatin–PVA–agar combinations successfully simulate porcine fatty tissue in electrosurgical applications [49], while sodium alginate (SA) and gelatin mixtures demonstrate potential for replicating the nonlinear mechanical properties of brain tissue [50, 51]. Because gelatin‐based phantoms typically have a limited shelf life of about 1 month, researchers add preservatives such as sodium benzoate or potassium sorbate (KS) to extend usability [52–55].

The choice of phantom materials depends on the anatomical region being simulated and the required properties. Head phantoms for EEG system validation and electrode testing vary significantly in material composition, electrical characteristics, anatomical accuracy, SNR performance, and durability. Textile‐based phantoms offer lightweight construction, resistance to degradation, and prolonged operational lifespan. Their electrical conductivity remains stable with minimal frequency dependence, while providing high initial SNR and excellent measurement reproducibility [56]. In contrast, gelatin‐ or agar‐NaCl‐based phantoms replicate the electrical and dielectric properties of human tissues more closely and allow adjustable frequency‐dependent behavior. Layered structures enhance anatomical realism, and realistic noise profiles facilitate precise assessment of electrodes and devices. Nevertheless, these phantoms remain constrained by water loss and microbial degradation, limiting their operational lifespan [57–60].

In our previous study [61], we explored the generation of artificial EEG signals using a mechanical vibration–based system where motor‐driven internal beams produced frequency‐specific waves. While that approach successfully demonstrated signal acquisition, it revealed limitations regarding environmental noise and the damping effects of rigid materials. To overcome these challenges and achieve superior bioequivalence, this study transitions from mechanical excitation to a material‐centric optimization approach. Gelatin‐based composites, enriched with SA and PVA, were developed by systematically varying graphite and salt ratios to precisely tune electrical conductivity and stabilize mechanical behavior. The methodology was validated using both RHSP and HHP. Consequently, this study focuses on the design and fabrication of anatomically accurate single‐layer conductive polymer‐based skull phantoms, replicating the electrical properties of biological tissues. These phantoms aim to provide a robust and reproducible platform for the development and optimization of EEG electrodes and headcaps.

## 2. Materials and Methods

### 2.1. Materials

Gelatin (220 Bloom value), PVA (1.19–1.31 g/cm^3^), SA (E401), graphite powder (160 × 50 μm), and KS (E202) were purchased from Öz Yaldız Chemical Materials Ind. Trade. Co., Ltd. (in Turkey). Reduced graphene oxide nanopowder thermochemical reduction dispersion (0.25 mg/mL) and silver nanopowder (100 nm) were purchased from Hazerfen Chemicals, Materials and Energy Technologies Industry and Trade Inc. (in Turkey). The above materials have not undergone additional processing prior to utilization.

The gelatin‐based phantoms were cast into cranial molds fabricated by fused deposition modeling (FDM). The molds were fabricated using a Creality CR‐10 Smart FDM printer, set to a printing temperature of 210°C, a bed temperature of 60°C, and a speed of 100 mm/s. The PLA filament was acquired from Porima Polymer Technologies Inc. in Turkey. A magnetic stirrer hot plate from the Isolab brand was utilized. The resistances of the generated phantoms were assessed utilizing a Hantek 1832C portable LCR meter. Subsequent to the fabrication of the gelatin‐based half skull, it was stimulated using an MFG‐6010 10 MHz signal generator. Artificial signals were obtained via an 8‐channel Cyton Biosensing Board EEG device. The team procured the biosensing board, which comprises 16 channels for acquiring artificial EEG brain waves, from OpenBCI [38]. Simulations of brain activity based on artificially generated signals were conducted utilizing the low‐resolution electromagnetic tomography (LORETA) program [62].

### 2.2. Preparation of Phantom

In developing a realistic skull phantom, the gelatin‐based composite phantoms were formulated using gelatin (G), NaCl, reduced graphene oxide (rGO) solution, silver nanopowder (Ag), graphite powder (Gr), PVA solution (PVA), SA solution (SA), and PVA/SA solutions. Materials were combined at varying concentrations, mixing durations, and volumes, resulting in 116 tissue‐mimicking RHSPs across six separate series, as detailed in Supporting Description, Table S1. All solutions were prepared with deionized water using a magnetic stirrer, and all experiments were conducted under identical laboratory conditions at room temperature.

PVA and SA, widely employed in tissue engineering, were incorporated to improve the mechanical properties of the phantoms, thereby making them more analogous to biological tissues. NaCl, rGO, Ag, and Gr were added to achieve the desired electrical conductivity. Considering the importance of gelatin and salt concentrations, Series 1 examined the effect of NaCl concentration (0–20 g/100 mL of deionized water) and gelatin concentration (30%, 45%, and 60% w/v), mixed at 50°C and 800 rpm on electrical conductivity. Series 2 investigated the effects of mixing duration (5, 40, and 75 min) and solution volume. A 5‐min duration was selected for practicality and reduced material consumption, although it yielded slightly lower conductivity than 75 min.

**FIGURE 1:**
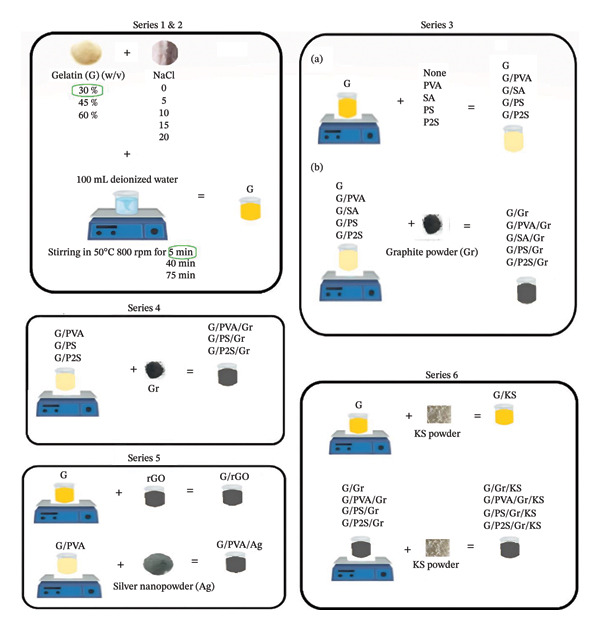
Production flowchart.

PVA and SA solutions (9% (w/v) and 1% (w/v), respectively) were prepared for phantom formulation and blended in volume ratios of 1:1 (v/v, PS) and 2:1 (v/v, P2S). In Series 3, phantoms were prepared by mixing G:PS and G:P2S at a 1:1 (v/v) ratio. However, due to freezing and mold‐release issues, the protocol was modified in Series 4, where G:PS and G:P2S were mixed at 3:1 (v/v) and 4:1 (v/v) ratios to optimize both conductivity and mechanical performance. Gr was incorporated into Series 3, 4, and 6 phantoms at ratios of 3:0, 3:2, and 3:4 (G:Gr) (w/w), respectively. Series 5 combined rGO and PVA/Ag with G at a 7:1 ratio (G:rGO and G:PVA/Ag, v/v), while the inclusion of KS in Series 6 enhanced shelf life. Figure 1 illustrates the complete production process.

**FIGURE 2:**
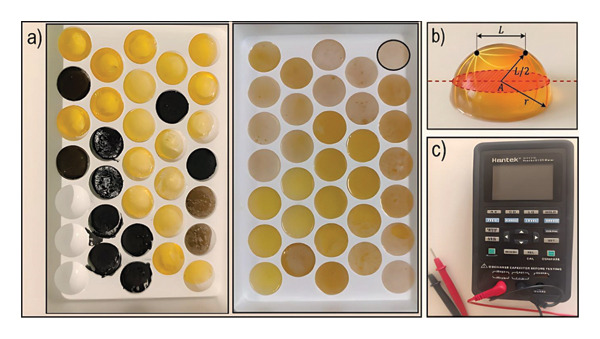
RHSP: (a) 1st and 2nd production series phantoms; (b) hemispherical geometric model; and (c) LCR meter.

The RHSP molds were fabricated using an FDM 3D printer, each containing hemispherical cavities with a diameter of 25 mm and a height of 12.5 mm (Figure 2). To investigate the effect of volume on conductivity, RHSP phantoms with volumes of 2, 4, and 5 mL were produced using these molds. For each of the 116 compositions, a minimum of three phantoms were created, which maintained stability for 5–6 days when stored in Ziploc bags. The measurement accuracy was verified using calibrated reference resistors prior to the experimental series.

Electrical resistance measurements were conducted utilizing an LCR meter (Hantek 1832C) in a conventional two‐probe arrangement. To determine the appropriate test frequency for obtaining a direct‐current (DC)‐like resistance value, a frequency sweep from 100 Hz to 1 MHz was initially performed on representative samples. The results indicated a frequency‐independent plateau in the measured impedance magnitude (|*Z*|) between 500 Hz and 5 kHz, confirming minimal electrode polarization effects and dominant ohmic behavior. This plateau signifies that the measured value within this range accurately represents the material’s intrinsic conductivity (σ). Since the propagation of low‐frequency currents (such as EEG signals) is governed by this fundamental σ, a fixed frequency of 1 kHz, centered within the ohmic plateau, was selected for all subsequent comparative measurements to ensure reliable and application‐relevant characterization. A consistent, minimal manual contact force was applied by a single operator to ensure stable probe contact without deforming the sample, and this standardized technique was maintained for all measurements. The probes were delicately placed against the hemispherical surface with a fixed center‐to‐center spacing (*L*) of 20 mm, using an AC test signal of 1 Vrms. To mitigate the effects of local heterogeneities—such as entrapped air bubbles or the enhanced surface roughness from graphite powder—which could influence resistance, measurements were taken at five distinct surface spots on each phantom over a 7‐day period. The arithmetic mean of these five measurements (*R*) was used for further analysis to diminish the impact of local variations.

The electrical conductivity (σ) of all phantoms was computed from the mean resistance (*R*) using the fundamental relation (1) [63]. For the hemispherical geometry, determining the effective cross‐sectional area (*A*) for the current flow required a geometric model. We modeled the current‐carrying cross section as the area of the circular disc that resides in the plane midway between the two probes and is contained within the hemisphere’s boundary (Figure 2(c)). This area is calculated using (2), where *r* is the hemisphere radius (12.5 mm) and *L* is the probe spacing (20 mm), yielding an effective area of approximately 95 mm^2^ for all phantoms. This consistent, geometrically derived model enables a fair comparison of the intrinsic conductivity across all compositions. Finally, the mass of each phantom was evaluated over the same 7‐day period to assess stability

(1) σ=LA×RS/m,

(2) A=πr2−L22.

### 2.3. Phantom Characterization via Experimental and Computational Methods

Based on the experimental data from the RHSP study, a regression model was developed using Python to identify the composition that most accurately replicates the electrical conductivity of soft tissues in the HHP. Figure 3 illustrates the system constructed for the HHP, which simulates soft tissue conductivity. The molds used for HHP fabrication [64] were produced via 3D printing. The selected composition, determined from the regression model (details of the regression model are provided in (2), below), was subsequently cast into half‐skull‐sized molds to construct the HHP. The electrical conductivity of the constructed skull phantom was assessed and confirmed to fall within the specified range. Probes connected to a signal generator were placed inside the phantom to deliver controlled electrical signals, while probes linked to the OpenBCI EEG board were positioned on the phantom’s surface to capture signals for visualization in the OpenBCI GUI. The signal generator was configured to deliver sinusoids.

FIGURE 3Measurement setup: (a) schematic illustration; (b) actual.

**Figure:**
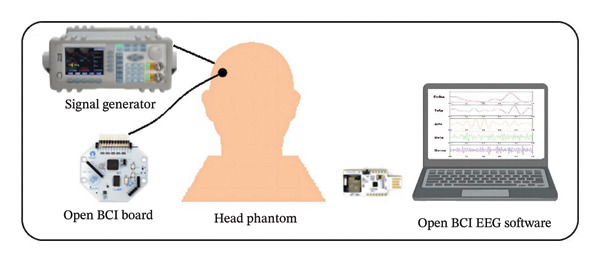
(a)

**Figure:**
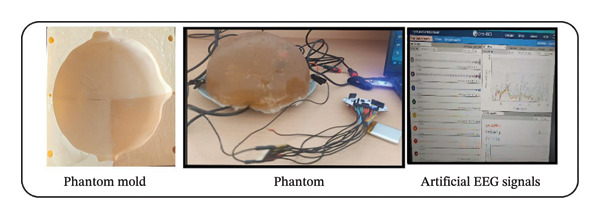
(b)

## 3. Results

### 3.1. Electrical Conductivity of Phantom

The electrical conductivity of all phantoms was determined using (1) to identify values closest to the volumetric conductivity of soft tissues. The reproducibility of the electrical measurements for each phantom composition was quantitatively assessed by calculating the standard deviation from the five‐point measurements performed on each sample. These standard deviations, representing the measurement precision, are visually presented as error bars in Figures 4(a), 4(b), 4(c), 4(d), and 4(e). The relatively small magnitude of these error bars across all series confirms the high repeatability of both our fabrication protocol and the electrical measurement technique. In Series 1, we observed that conductivity—controlled by gelatin and salt concentrations—plateaued when salt exceeded 10 g/100 mL and gelatin surpassed 45% (w/v) (Figure 4(a)). Series 2 results showed that 30% (w/v) gelatin phantoms containing 5 g/100 mL of NaCl reached maximum conductivity after 75 min of mixing, whereas phantoms with 10 g/100 mL of NaCl achieved comparable conductivity after only 5 min, matching the values obtained after 40 and 75 min (Figure 4(b)). These observations confirmed that gelatin–salt solutions effectively replicate soft tissue conductivity. To investigate the effect of G:Gr ratios (3:0, 3:2, and 3:4 (w/w)) and salt concentration on electrical conductivity, we prepared Series 3 phantoms (Figure 4(c)). We found that adding graphite markedly increased conductivity in phantoms containing 5 g/100 mL or 10 g/100 mL of NaCl, and higher salt levels consistently enhanced conductivity across all graphite‐containing formulations. Based on these results, we selected a G:Gr ratio of 3:2 (w/w) for subsequent studies. Despite achieving optimal electrical performance, graphite‐containing phantoms exhibited mold adhesion, so we incorporated PVA and SA to reduce weight and improve mechanical stability. We also evaluated different PVA/SA ratios, revealing that SA contributed minimally to total weight. In Series 4, we addressed freezing and mold‐release challenges by adjusting G:PVA/SA ratios to 3:1 and 4:1 (v/v), successfully improving mold handling.

FIGURE 4Electrical conductivity: (a) according to different salt and gelatin ratios, (b) the effect of mixing times of 5, 40, and 75 min for 30% (w/v) gelatin‐based phantoms with 0 and 10 g/100 mL of NaCl, (c) according to the G:Gr ratio (w/w), (d) based on KS content, (e) the electrical conductivity of the phantoms, and (f) phantoms (1) density comparisons based on salt and gelatin ratios (%w/v), (2) density comparisons with literature values, and (3) average mass difference after a 7‐day measurement.

**Figure:**
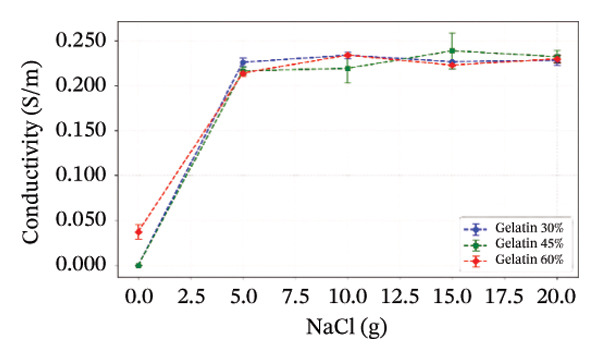
(a)

**Figure:**
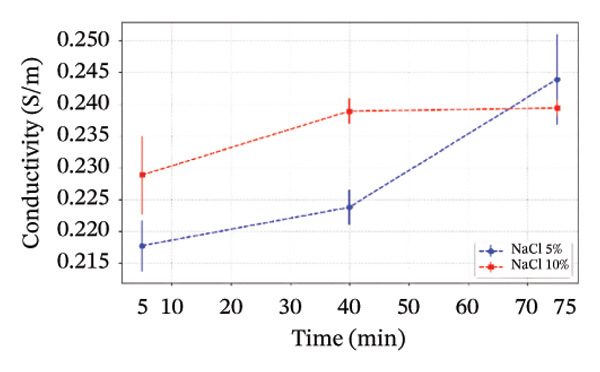
(b)

**Figure:**
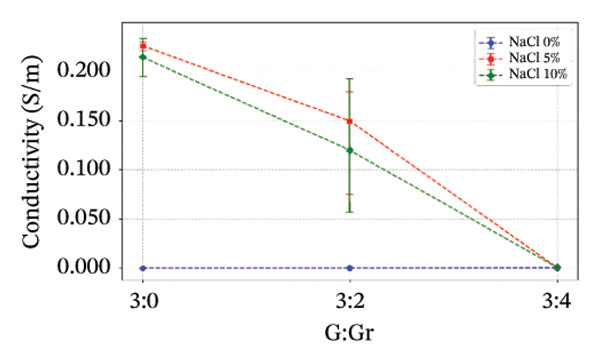
(c)

**Figure:**
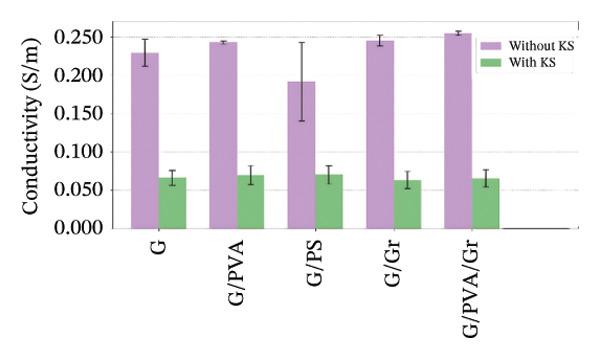
(d)

**Figure:**
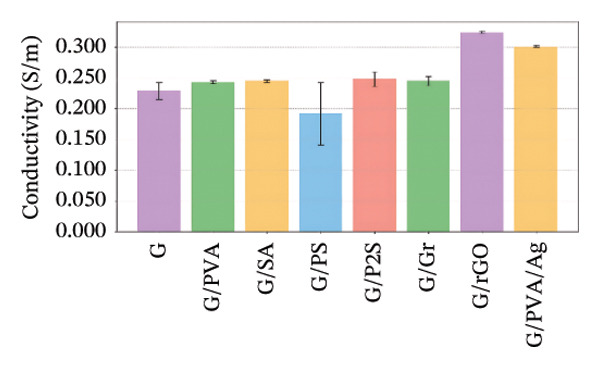
(e)

**Figure:**
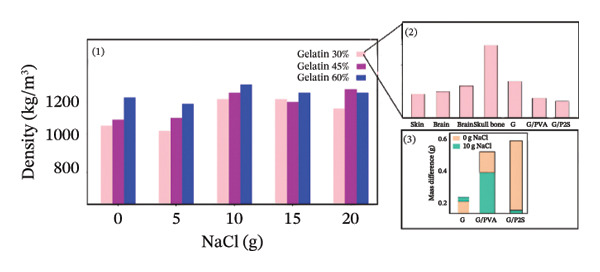
(f)

In Series 5, we overcame adhesion and fragmentation limitations by using rGO and PVA/Ag, producing phantoms with high electrical conductivity. Among all series, the G/rGO phantom exhibited the highest conductivity. In Series 6, we incorporated KS to extend phantom shelf life (Figure 4(d)), resulting in phantoms with an average lifespan of 50 days. Although resistance values slightly increased compared to earlier series, these KS‐containing phantoms maintained electrical properties suitable for simulating low‐conductivity tissues such as internal air, external air, and adipose tissue (Table 1). Detailed results and comparisons with experimental data are provided, with Figure 4(e) summarizing the conductivity of all phantom formulations.

**TABLE 1:** A list of the compositions of the produced phantoms and the corresponding biological tissues.

G (%w/v)	NaCl (g/100 mL)	G:PVA (v/v)	G:PS (v/v)	G:P2S (v/v)	G:Gr (w/w)	G:rGO (v/v)	G:PVA/Ag (v/v)	KS (g/100 mL)	Conductivity (S/m)	Tissue
30	5	1:1	—	—	3:2	—	—	—	0.0023	Air Internal
30	5	—	—	1:1	—	—	—	—	0.0027	
45	—	—	—	—	—	—	—	—	0.0020	

30	10	—	3:1	—	—	—	—	2	0.0702	Dura, Skull soft bone, Skull hard bone, Muscle, Fat, Hypothalamus, Liver, Nerve, Tooth, Cortical bone
30	10	—	—	—	—	—	—	2	0.0660	
60	—	—	—	—	—	—	—	—	0.0294	
60	5	—	—	—	—	—	—	—	0.0455	

30	5	—	3:1	—	3:2	—	—	—	0.1352	Brain white matter, Cerebellum, Heart, Kidney, Lung
30	10	—	3:1	—	—	—	—	—	0.1420	
30	5	—	3:1	—	—	—	—	—	0.1414	
30	10	—	—	3:1	3:2	—	—	—	0.1428	

30	5	—	—	—	—	—	—	—	0.2177	Soft Tissue, Eye lens, Bladder
30	10	—	—	—	—	—	—	—	0.2288	
30	10	—	—	3:1	—	—	—	—	0.2580	
30	10	—	—	4:1	—	—	—	—	0.2460	
30	10	—	4:1	—	—	—	—	—	0.1950	
30	10	—	3:1	—	—	—	—	—	0.1923	
45	15	—	—	—	—	—	—	—	0.2384	
30	5	—	3:1	—	—	—	—	—	0.2514	
30	10	3:1	—	—	—	—	—	—	0.2409	

30	10	—	—	—	—	7:1	—	—	0.3240	Scalp, Skin, Brain, Muscle, Brain gray matter, Tongue, Ligament, Diaphragm, Trachea, Tendon, Pancreas
30	10	—	—	—	—	—	7:1	—	0.3024	

### 3.2. Density of Phantom

**FIGURE 5:**
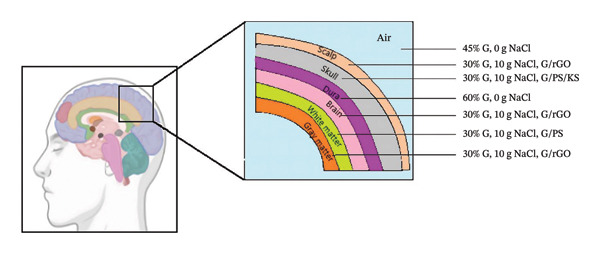
Phantom components suitable for head layers.

Phantom densities were calculated based solely on their gelatin and salt compositions, considering gelatin concentrations of 30%, 45%, and 60% w/v and NaCl amounts of 0, 5, 10, 15, and 20 g/100 mL of deionized water. These calculated densities were then compared with the reported densities of human tissues, including skin [65, 66], brain, and skull bone [67] (Figures 4(f) (1) and 4(f) (2)). The results indicate that the densities of gelatin‐based phantoms closely approximate those of human skin and brain tissue. Figure 4(f) (3) illustrates the mass alterations of the phantoms following a 7‐day measuring period. The average mass loss values of all phantoms in the studies ranged from 0.10 to 0.60 g. The mean masses of the G, G:PVA 3:1 (v/v), and G:P2S 3:1 (v/v) phantoms, of identical size, were 3.58, 3.20, and 3.61 g for phantoms with 0 g/100 mL of NaCl and 4.17, 3.40, and 3.07 g for those with 10 g/100 mL of NaCl, respectively. The gelatin‐based phantoms devoid of PVA and SA mixes exhibited the greatest mass, irrespective of the salt concentration. The incorporation of both salt and P2S rendered the phantoms less dense. Based on the findings from Series 1–6, Figure 5 illustrates the resulting phantom compositions designed to mimic the human skull layers.

The electrical conductivity of human head tissues exhibits significant variability in the literature due to their anisotropic nature and intersubject physiological differences. For instance, reported values range from 0.100 S/m to 0.333 S/m for gray matter [60, 68], 0.060 to 0.142 S/m for white matter [60, 68], and 0.30 to 0.97 S/m for whole‐brain tissue [30, 41, 60]. Studies on anisotropic mouse head phantoms further support these ranges, reporting a brain conductivity of 0.33 S/m, which aligns closely with human values [41]. Similarly, skull hard bone conductivity varies between 0.02 S/m and 0.09 S/m [30, 60, 68], while soft bone values range from 0.04 S/m to 0.06 S/m [30, 60]. The dura mater is typically reported around 0.06 S/m [60], and scalp conductivity shows considerable variation, spanning 0.100–0.64 S/m [30, 60, 68]. Overall, electrical conductivity across head tissues can vary over three orders of magnitude (10^−3^ to 1 S/m) due to structural anisotropy and individual metabolic activity [69].

As shown in Table 1, the fabricated head phantoms closely match these biological conductivity values, demonstrating their efficacy in emulating the electrical properties of actual human tissues.

To ensure a homogeneous distribution of KS throughout the phantom, we first codissolved gelatin and KS in deionized water before adding the remaining components. Consequently, all electrical conductivity values reported herein are derived from solutions prepared with 100 mL of deionized water for both NaCl and KS. A ratio of G:P2S at 3:1 (v/v) yielded an electrical conductivity comparable to that of soft tissue. Phantoms containing G:PS and G:P2S in a 4:1 (v:v) ratio attained conductivity levels comparable to those of cranial tissue. The G:rGO phantom exhibited electrical conductivity values appropriate for tissues including the scalp, skin, brain, muscle, gray matter, tongue, ligament, skull, trachea, tendon, and pancreas. A phantom with the electrical conductivity of the scalp, created through casting, is available in the literature for testing EEG equipment [3]. A practical scalp phantom can be created, utilizing our G:rGo composite formulation instead of casting.

### 3.3. Mathematical Study of Phantoms

We developed a regression model (equation (3)) in Python using electrical conductivity data from 2, 4, and 5 mL of phantom experiments. The model yielded a mean‐squared error (MSE) of 0.0070, indicating high predictive accuracy and a coefficient of determination (*R* ^2^) of 0.4041. This initial *R* ^2^ value signifies that 40.41% of the variance in the dependent variable is explained by the model. Subsequent evaluation with a decision tree regressor improved the *R* ^2^ to 0.5441, accounting for 54.41% of the target variance, with a mean absolute percentage error (MAPE) of 22.61%.

Guided by these predictions and experimental data, we fabricated a half‐ HHP. Although materials like rGO and Ag can produce tissues with conductivities closer to target values (e.g., 0.3240 S/m for skin, brain, and scalp), their cost is prohibitive for a phantom of this volume. Therefore, we selected a more economical gelatin‐based phantom with a G:P2S ratio of 3:1 (v/v), which exhibits a conductivity of 0.2580 S/m. Despite a 20.37% discrepancy from the target value, this variance is sufficiently minor to justify the use of G/P2S for large‐scale HHP production

(3) y=0.02050.00040.00850.04740.052520.07120.03820.01130.20980.23920.21570.0267−∗G+∗NaCl−∗PVA−∗PS+∗PS+∗SA+∗Gr+∗G/rGO+∗G/PVA/AgmL−∗KS+∗Volume.

### 3.4. Artificial EEG Signals

**FIGURE 6:**
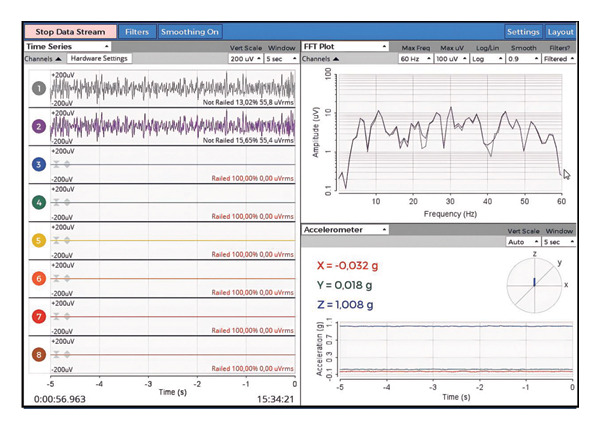
EEG signal from the OpenBCI board.

EEG measures electrical impulses produced by the brain using electrodes placed on the scalp. A prevalent method for categorizing EEG waves involves the analysis of the frequency spectrum. Artificial EEG brain waves are produced by applying sine waves of specified frequency and amplitude to the phantom via signal generators, resulting in artificial EEG signals [70, 71]. The spectra indicate that the delta wave ranges from 0.5 to 4 Hz, the theta wave from 4 to 7 Hz, the alpha and mu waves from 8 to 12 Hz, the sigma wave from 12 to 16 Hz, the beta wave from 13 to 30 Hz, and the gamma wave is 30 Hz and higher [72, 73]. This investigation involved the placement of three dipole wires within the HHP phantom to simulate neural activity. The American Academy of Sleep Medicine (AASM) recommends that three electrodes are enough for the development of polysomnography (PSG) EEG devices [74]. Consequently, three dipole wires were integrated into the phantom, and in conjunction with a reference electrode, two recording electrodes were linked to the OpenBCI Cyton board. In accordance with prior methodologies in the literature, a 10‐Hz sine wave [3, 56] with a 1‐V amplitude [75] was generated using a signal generator, introduced into the phantom, and false EEG signals were produced through the Cyton board. The phantom signal parameters were modified to replicate EEG activity within the alpha wave spectrum. The data obtained via the OpenBCI GUI validated the production of synthetic alpha waves. Figure 6 illustrates the frequency and amplitude characteristics of the alpha waves, derived from fast Fourier transform (FFT) analysis, and the real‐time signal acquisition process can be observed in Video S1 (in Supporting Description).

To validate the phantom, a synthetic sine wave (200‐mV peak‐to‐peak, 100‐mV maximum, −100‐mV minimum, 10‐Hz frequency) was produced by the signal generator and captured via the Cyton board. SNR was employed to assess the quality of signals broadcast to and received from the phantom, as it indicates the strength of the detected signal in relation to background random noise [56, 76]. To compute the SNR, a 30‐s baseline recording was acquired with the phantom source deactivated to assess the noise level. Subsequently, upon the injection of the 10‐Hz signal, the root‐mean‐square (RMS) values for both the signal and the noise were calculated, and the SNR was determined using (3). The average SNR across all channels was determined to be 12.52 ± 2.1 dB using this method. Recordings were executed using the Cyton board set at a sampling rate of 250 Hz, with a 50/60‐Hz notch filtering activated. The results indicate that the EEG head phantom, in conjunction with the Cyton board, delivers adequate signal quality to replicate human alpha wave activity

(4) SNRdB=10logPeak to Peak Voltage SignalPeak to Peak Voltage Noise.

**FIGURE 7:**
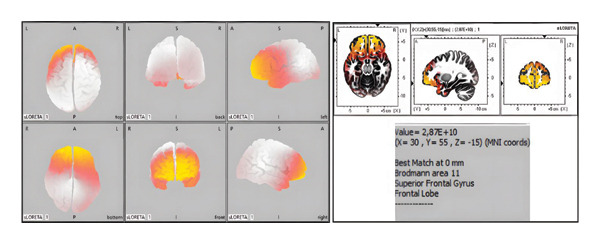
Visualization of the HHP prepared with G/P2S using the LORETA simulation.

The data obtained from the OpenBCI board were processed and transmitted to the LORETA program, facilitating the three‐dimensional reconstruction of brain electrical activity distribution. Furthermore, LORETA is a pioneering instrument in neuroimaging characterized by great temporal resolution [77]. In the LORETA brain simulation, we injected sine waves into the frontal region of the HHP and observed that the resulting activity was concentrated in the frontal area from all viewing angles (Figure 7).

## 4. Discussion

This study successfully developed a versatile and tunable suite of gelatin‐based phantom materials capable of mimicking the wide spectrum of electrical conductivity properties found in human cranial tissues. Our findings underscore the critical trade‐offs inherent in phantom design, necessitating a careful balance among electrical performance, mechanical properties, manufacturing efficiency, cost‐effectiveness, and durability.

This methodology was implemented to develop a versatile suite of materials. In contrast to the multilayer, reconfigurable phantoms that offer high electrical realism [26], or the complex anatomically accurate 3D‐printed models [28–30], this work presents a single‐layer, conductive polymer‐based approach. Our primary contribution lies in its simplicity, reproducibility, and focus on cost‐effective fabrication for initial electrode–skin interface testing. Compared to gelatin‐ or agar‐based phantoms that closely mimic frequency‐dependent properties but suffer from short shelf‐life [57–60], our polymer‐based phantom aims for improved durability and operational stability. Furthermore, while textile‐based phantoms offer excellent durability [58], they may lack anatomical accuracy; our work bridges this gap by combining anatomical shape with a stable conductive polymer.

Guided by this design philosophy, we successfully fabricated a total of 116 small‐scale RSHPs and one full HHP across six series, yielding a broad range of reproducible electrical conductivity values. The optimized formulation of 30% (w/v) G and 10 g/100 mL of NaCl served as a foundational base, offering an optimal balance between conductivity and practical handling. Refinements in mixing duration revealed that while 75 min yielded the highest conductivity, a 5‐min mix for the 10 g/100 mL NaCl formulation provided stable and sufficient electrical performance, emphasizing efficiency and scalability for future production. The incorporation of Gr substantially enhanced conductivity, with a 3:2 (w:w) G:Gr ratio producing the highest values. However, these composites introduced mold adhesion issues, which were significantly mitigated by integrating PVA and SA solutions. These additives not only improved material cohesion but also reduced the weight and bulk of the phantoms, enhancing their practicality for large‐scale models such as the HHP. While contact force can influence single‐point measurements on rough surfaces, the use of a standardized technique and multipoint averaging mitigated its impact on the reported mean conductivity values.

Resistance to microbial growth was another critical factor; incorporating KS powder increased the average phantom lifespan to 50 days, rendering these formulations appropriate for prolonged experimental use. Although KS increased electrical resistance, this property makes it particularly suitable for simulating low‐conductivity tissues such as air‐filled cavities.

It is important to contextualize the role of the regression model (Equation (3)) developed to analyze the small‐scale phantom data. While the model provided valuable qualitative insights—identifying the direction and relative strength of component effects on conductivity (e.g., the strong negative influence of P2S and PVA)—its predictive power, as indicated by *R* ^2^ values of 0.40–0.54, is quantitatively limited. This is an expected reflection of the high‐dimensional complexity and inherent nonlinear interactions within multicomponent polymer systems, rather than a failure of the approach. The model’s primary utility was not to achieve high‐fidelity numerical prediction but to serve as a robust exploratory tool. It effectively narrowed the vast composition space and guided our empirical optimization toward promising regions, ultimately leading to the identification of the 3:1 G/P2S formulation as a viable candidate for the HHP when balanced against practical constraints like cost and stability.

A key limitation of this initial study is the focus on electrical characterization over mechanical property replication. Future iterations will incorporate mechanical testing to fully assess tissue mimicry. However, the demonstrated electrical properties and anatomical form factor already provide a valuable tool for the standardized testing of electrode contact impedance and baseline performance in a geometrically realistic setting.

Although advanced materials such as rGO and Ag produced the highest conductivity values, their high cost limits large‐scale use. Importantly, the resulting phantom demonstrated conductivity similar to human scalp, indicating its potential as a noncasting alternative reported in the literature. This cost–performance trade‐off led to the selection of a G:P2S ratio of 3:1 (v:v) for HHP construction, which, despite a ≈20% deviation from ideal scalp conductivity, provided an optimal compromise for developing a functional, scalable, and cost‐effective model.

The successful fabrication and validation of the HHP demonstrate that small‐scale RSHP data can be effectively extrapolated to guide the production of larger, anatomically realistic models. Electrophysiological validation confirmed the HHP’s functional utility: it successfully propagated synthetic alpha waves from an external stimulator, and subsequent LORETA‐based source localization accurately reconstructed the expected activity in the frontal region. This result confirms the HHP’s applicability as a reliable testbed for the EEG electrode validation, source localization algorithms, and imaging system calibration, free from the biological variability inherent in human studies.

In conclusion, this work provides a robust methodological framework and material toolkit for developing realistic, tunable, and durable tissue‐equivalent phantoms. The insights gained into material properties, trade‐offs, and scaling relationships represent a valuable foundation for future research in neurodiagnostics and electromagnetic simulation.

## 5. Conclusions

This study demonstrated the viability of developing gelatin‐based phantoms that closely replicate the electrical conductivity characteristics of real cranial tissues. An economical and easily producible HHP was successfully formulated utilizing a 3:1 (v/v) blend of G and P2S, which attained the requisite conductivity and significantly resolved mold adhesion problems. The HHP‐generated artificial EEG brain waves validated its capability for physiological assessments and brain activity simulations. The production formulae outlined in this work are adaptable for the creation of multilayer phantoms for ultrasonography and skin phantoms for EMG and EKG measurements. Subsequent research will concentrate on incorporating these phantoms into EMG and EKG systems to replicate muscle and cardiac tissues. Furthermore, examining the viscoelastic features of the phantoms will yield significant insights, facilitating comparisons with the viscoelastic qualities of the brain and other tissues or organs.

**Nomenclature**
BCI: Brain–computer interface
CT: Computed tomography
ECG: Electrocardiography
EEG: Electroencephalography
EIT: Electrical impedance tomography
EMG: Electromyography
FDM: Fused deposition modeling
FFT: Fast Fourier transform
G: Gelatin solution
HSP: Human head phantom
KS: Potassium sorbate (potassium (2E,4E)‐hexa‐2,4‐dienoate)
LORETA: Low‐resolution electromagnetic tomography
MAPE: Mean absolute percentage error
MRI: Magnetic resonance imaging
MSE: Mean‐squared error
PLA: Polylactic acid
PVA: Polyvinyl alcohol
PVA/Ag: PVA solution/silver nanopowder
rGO: Reduced graphene oxide nanopowder thermochemical reduction dispersion (0.25 mg/mL)
RHSP: Rat head size phantom
SA: Sodium alginate
SAR: Specific absorption rate
SNR: Signal‐to‐noise ratio

## Author Contributions

Conceptualization, E.N.S. and G.Ç.; methodology, E.N.S. and G.Ç.; validation, E.N.S., G.Ç., and M.R.U.; formal analysis, E.N.S.; investigation, E.N.S. and G.Ç.; resources, E.N.S., G.Ç., and M.R.U.; data curation, E.N.S. and G.Ç.; writing–original draft preparation, E.N.S. and G.Ç.; writing–review and editing, E.N.S., G.Ç., and M.R.U.; visualization, E.N.S.; supervision, M.R.U.; project administration, M.R.U.; funding acquisition, M.R.U. All authors agree to be accountable for the content and conclusions of the article.

## Funding

This study was supported by the Scientific Research Projects Coordination Unit of Süleyman Demirel University under project number FDK‐2022‐8789.

## Conflicts of Interest

The authors declare no conflicts of interest.

## Supporting Information

The supporting information provides additional details supporting the findings of this study. Supporting Information 1 includes Table S1, which presents the production parameters, material compositions, and dimensional specifications of the rat head size phantom (RHSP) and human head phantom (HHP). These data ensure the reproducibility of the developed gelatin‐based phantom models. Video S1.mov demonstrates the real‐time acquisition of EEG signals using the OpenBCI system, illustrating signal stability and responses to simulated neural activity.

## Supporting information


**Supporting Information** Additional supporting information can be found online in the Supporting Information section.

## Data Availability

Data are available from the corresponding author upon request.
