# Supplementary material for: Gelatin‐Based Head Phantoms: A Practical Guide for Artificial Brain Signal Research
Source: Int J Biomater. 2026 Apr 29;2026:2255123. doi: 10.1155/ijbm/2255123 (PMC13126086; doi:10.1155/ijbm/2255123)
Supplement: Supplementary file 1 — Supporting Information Additional supporting information can be found online in the Supporting Information section. [file IJBM-2026-2255123-s001.zip › supporting_information_1.docx]

**GELATIN-BASED HEAD PHANTOMS: A PRACTICAL GUIDE FOR ARTIFICIAL EEG SIGNAL RESEARCH**

Elif N. Selçuk*, Gökçenur Çakmak Keçelioğlu, Mustafa R. Usal

**SUPPLEMENTARY MATERIALS**

**The file includes:**

Table S1. Production parameters of rat head size phantom (RHSP) and human head phantom (HHP)

**Other supplementary information for this manuscript includes:**

Video S1: EEG signals acquired from phantoms using OpenBCI.

**TABLES**

Table S1. Production parameters and corresponding electrical conductivity values of RHSP and HHP.

Table S1 (continued).

Table S1 (continued).

Table S1 (continued).

The percent weight/volume (% w/v) values used in this study are 9% (w/v) for the PVA solution and 1% (w/v) for the SA solution. The PVA solutions were referred to as PS for the 1:1 (v:v) ratio and P2S for the 2:1 (v:v) ratio.

*The values provided are for productions made with 100 mL of deionized water.
